# Understanding Non-Small-Cell Lung Cancer: Biology, Therapeutics and Drug Resistance

**DOI:** 10.3390/biomedicines14010193

**Published:** 2026-01-15

**Authors:** Pankaj Ahluwalia, Ravindra Kolhe, Mumtaz Rojiani

**Affiliations:** 1 Department of Pathology, Medical College of Georgia, Augusta University, Augusta, GA 30912, USA; 2 Department of Neuroscience and Experimental Therapeutics, Penn State College of Medicine, Hershey, PA 17033, USA; 3 Penn State Cancer Institute, Hershey, PA 17033, USA

Lung cancer remains the leading cause of cancer-related deaths globally, with around 2.5 million new cases and approximately 1.8 million deaths in 2022 [1]. Non-small-cell lung cancer (NSCLC) accounts for 80–85% of all lung cancer diagnoses and includes adenocarcinoma, squamous cell carcinoma and large-cell carcinoma subtypes [2]. Over the past three decades, progress in cancer biology is increasingly translated into clinical benefit through improved diagnostics and therapies [3]. However, recent American Cancer Society estimates report 5-year relative survival rates of 67% (localized), 40% (regional), and 12% (distant) for NSCLC [4]. Despite significant improvements in screening, molecular diagnosis, and therapeutic advances, outcomes for advanced disease remain poor. There is a continued need to develop new tools for early detection, patient stratification, and durable treatment response.

Over the last couple of decades, two major therapeutic advantages have yielded significant benefits to lung cancer patients: targeted therapy (tumor genotyping based on next-generation sequencing) and immunotherapy (immune checkpoint blockade). Both approaches are now routine components of NSCLC management. For example, randomized evidence has established checkpoint inhibition in the post-chemoradiation setting as standard care in unresectable stage III disease. Further, third-generation EGFR inhibition with broader efficacy has improved outcomes in EGFR-mutant NSCLC [5]. Yet, a central challenge persists with these approaches: most patients either do not respond or develop acquired resistance, limiting long-term disease control and reinforcing the need for better understanding of the underlying biology and improved prognostic and predictive biomarkers [6].

To contextualize the field’s emphasis on therapeutic resistance, a PubMed ‘Results by Year’ trend analysis using a resistance-focused NSCLC query showed a ~14.8-fold increase in annual publications from 133 in 2000 to 1964 in 2024 (Figure 1), consistent with the growing need to address relapse and treatment failure.

To further highlight the research in this field, this Special Issue was designed to attract contributions spanning NSCLC biology, biomarkers, and therapeutic strategies. The five papers published in this Special Issue span pharmacogenomics of platinum therapy, interpretation of PD-L1 after chemoradiation, non-coding RNA biology, extracellular vesicles and exosomes as biomarkers and therapeutic platforms, and fourth-generation EGFR inhibition and resistance mechanisms.

PD-L1 phenotyping is critical for checkpoint inhibitor selection in advanced NSCLC [7]. However, PD-L1’s prognostic value remains inconsistent and is complicated by assay variability, timing, and treatment-induced modulation. In this Special Issue, Wagner et al. (contribution 1) analyzed stage III NSCLC patients treated with chemoradiotherapy and investigated whether lack of PD-L1 expression confers a favorable prognosis [8]. The authors conclude that the median OS and PFS did not differ significantly by PD-L1 < 1% versus ≥1%. These findings support a cautious interpretation of PD-L1 as a prognostic marker in the post-chemoradiation setting [9]. As biomarker research advances, improving the contextual interpretation of PD-L1 together with composite biomarkers (e.g., TMB, interferon-related expression signatures, ctDNA dynamics, spatial immune architecture, microbiome, circulating markers, etc.) will be essential for reliable and robust stratification [10].

In another study, Zou et al. (contribution 2) evaluated germline DNA repair polymorphisms in platinum-treated lung cancer [11]. Platinum-based chemotherapy remains central in NSCLC treatment, alone or in combination [12]. The authors evaluated polymorphisms in DNA repair genes in lung cancer patients treated with platinum-based chemotherapy and reported that the ERCC5 rs873601 variant was significantly associated with overall survival. This finding supports the potential clinical relevance of germline pharmacogenomics, particularly in DNA repair pathways, in stratifying platinum-treated patients [13].

Non-coding RNAs have emerged as key regulators in NSCLC progression, metastasis, and therapy response. Among these, circular RNAs (circRNAs) play a key role due to their stability, and diverse regulatory roles are increasingly recognized [14,15]. In this Special Issue, Yuan et al. (contribution 3) investigated hsa_circ_0092856, a circRNA derived from the eIF3a locus, and demonstrated its oncogenic effects in NSCLC models [16]. Using qRT-PCR and functional assays, they showed that hsa_circ_0092856 was highly expressed in NSCLC cells, and that knockdown inhibited proliferation, migration, and invasion, while overexpression produced the opposite phenotype. They propose a hsa_circ_0092856–eIF3a regulatory axis that may contribute to NSCLC progression.

Liquid biopsy is increasingly playing a key role in NSCLC management, yet key gaps remain in sensitivity and specificity, and clinical standardization across platforms. Extracellular vesicles (EVs), particularly exosomes, are emerging as an attractive biomarker source because they are relatively stable in circulation and carry nucleic acids and proteins that could be leveraged clinically. In the next contribution, Rahimian et al. (contribution 4) provide a comprehensive review of EVs and exosomes in lung cancer, from early detection and prognostic applications to targeted treatment approaches [17]. These developments strengthen the rationale for EV-based biomarkers to complement tissue profiling and ctDNA, particularly for longitudinal monitoring of patients [18].

At the precision oncology level, EGFR-mutant NSCLC illustrates both the promise and challenge associated with targeted therapies. Third-generation EGFR TKIs have improved outcomes and CNS disease control, yet resistance via EGFR C797S and diverse bypass pathways such as MET activation and phenotypic switching (including epithelial–mesenchymal transition features) remain a significant barrier to long-term control. This has driven the development of fourth-generation EGFR inhibitors and rational combination strategies. In this Special Issue, Fukuda et al. (contribution 5) evaluated the fourth-generation EGFR-TKI BI4020 and explored resistance mechanisms in vitro [19]. They reported that resistant in vitro models did not harbor secondary EGFR mutations but pointed to other non-EGFR-mutation-mediated resistance routes including MET amplification and EMT features. Together, these findings support biomarker-guided combinations targeting bypass pathways and phenotypic plasticity to improve durable response.

Collectively, the five contributions in this Special Issue showcase how advancements in NSCLC biology continue to generate insights that are increasingly crucial for patient care. These contributions emphasize the relevance of germline pharmacogenomics, the need for contextual interpretation of PD-L1, the emerging roles of non-coding RNAs and extracellular vesicles, and sustained efforts to address drug resistance. With continued advances in multiomics profiling, computational modeling, and clinically deployable biomarker platforms, we anticipate that the next decade could enable improved stratification, better resistance management, and more durable benefit across broader patient subsets. As the field advances rapidly, the domains of artificial intelligence, and machine learning are poised to provide substantial benefits, particularly in decision support and risk stratification [20]. Digital pathology and deep learning, alongside AI-driven radiomics, can support early detection, staging, and outcome prediction, particularly when integrated with other molecular markers [21]. Further, early quantum-assisted generative modeling approaches have yielded experimentally validated KRAS-targeting candidates, highlighting a transformative frontier for drug discovery [22]. Ultimately, the convergence of molecular biology, robustly validated biomarkers, and clinically grounded computational tools offers a promising path to reduce the morbidity and mortality of NSCLC.

## Figures and Tables

**Figure 1:**
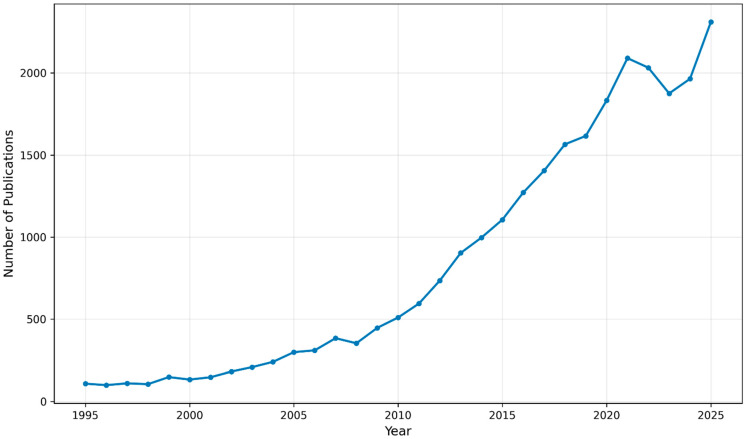
Trend analysis of PubMed-indexed NSCLC literature focused on treatment resistance and failure (1995–2025) accessed 14 December 2025.
